# Inertial measurement unit–based characterization of gait patterns in Asian elephants: clinically sound elephants and elephants with gait abnormalities

**DOI:** 10.3389/fvets.2026.1888434

**Published:** 2026-08-26

**Authors:** Siripat Khammesri, Kittichai Wantanajittikul, Kittikul Namwongprom, Narueporn Kittisirikul, Chatchote Thitaram, Niyada Thitaram, Janine L. Brown, Siriphan Kongsawasdi

**Affiliations:** 1 Department of Physical Therapy, Faculty of Associated Medical Sciences, Chiang Mai University, Chiang Mai, Thailand; 2 Department of Radiologic Technology, Faculty of Associated Medical Sciences, Chiang Mai University, Chiang Mai, Thailand; 3 Elephant Hospital, National Elephant Institute, Forest Industry Organization, Lampang, Thailand; 4 Center of Elephant and Wildlife Health, Animal Hospital, Faculty of Veterinary Medicine, Chiang Mai University, Chiang Mai, Thailand; 5 Faculty of Veterinary Medicine, Chiang Mai University, Chiang Mai, Thailand; 6 Center of Elephant, Wildlife and Companion Animals Research, Chiang Mai University, Chiang Mai, Thailand; 7 Center for Species Survival, Smithsonian National Zoo and Conservation Biology Institute, Front Royal, VA, United States

**Keywords:** Asian elephant, gait analysis, inertial measurement unit, kinematic parameters, gait abnormality

## Abstract

Objective gait assessment is important for detecting locomotor abnormalities in Asian elephants, but clinical interpretation remains constrained by the limited availability of species-specific gait data from clinically sound elephants and the practical constraints of conventional laboratory-based gait analysis. This study aimed to report preliminary observed values for IMU-derived gait parameters in clinically sound Asian elephants and to explore their application for characterizing abnormal gait patterns. Four inertial measurement unit sensors were attached bilaterally over the humerus and femur to record gait data during straight-line walking at a controlled speed. Ten clinically sound elephants were used to describe preliminary observed values, while four elephants with clinically identified abnormal gait patterns were evaluated individually through descriptive case comparison. Temporospatial parameters and duty factor (DF) were analyzed to characterize stance allocation, while cross-correlation coefficients (COF) of left–right vertical displacement waveforms were used to quantify bilateral vertical movement similarity. In clinically sound elephants, stride timing was generally comparable between sides, whereas stance-related parameters showed a consistent side-related pattern. Descriptive comparison with abnormal cases showed case-specific alterations in DF and COF. These findings provide preliminary observed values for IMU-based gait assessment in Asian elephants and support the complementary descriptive use of these parameters for characterizing stance allocation and bilateral vertical movement similarity. Larger clinical datasets are needed to establish clinically relevant thresholds and strengthen their clinical application.

## 1 Introduction

Assessing gait characteristics provides vital information about locomotor patterns and pathological deviations. Understanding normal gait is essential for interpreting abnormal findings, as gait abnormalities or asymmetries are typically recognized clinically as lameness. Such alterations may arise from pathological or dysfunctional interactions among muscles, tendons, bones, ligaments, joints, and components of the central or peripheral nervous system (1). Lameness scores or scales are widely used among domestic animals such as dogs (2–4), horses (5), and cattle (6), and represent subjective methods for assessing locomotor abnormalities that rely on visual appraisal by experienced clinicians. Despite widespread use, no standardized, species-specific scoring system has been validated for elephants, which limits clinical assessment in this species. Schiffmann et al. (7) developed a visual guide that relies on weight distribution, posture, compensatory mechanisms, joint deformities, and abnormal gait patterns as indicators of musculoskeletal disorders in zoo elephants. However, subjective lameness scoring is inconsistent and only moderately reliable across species and assessors, particularly when compared with objective or sensor-based gait analyses (8, 9).

Objective gait analysis provides quantitative insight into locomotor behavior and its underlying mechanics. Kinematics describes the spatial and temporal characteristics of movement, whereas kinetics focuses on the forces (10). In quadrupeds, and particularly in large species such as Asian elephants, gait evaluation must consider species-specific biomechanical constraints and behavioral adaptations. Conventional laboratory-based methods, such as optical motion capture, force platforms, and pressure walkways, offer high precision, repeatability, and reproducibility by minimizing external influences (11). However, the structural and spatial requirements of these systems make them impractical for large terrestrial mammals such as Asian elephants, where extreme body mass, ground reaction forces, flooring strength requirements, and safety considerations present substantial logistical and engineering challenges. In contrast, wearable sensor systems that can be directly attached to the body, including accelerometers, gyroscopes, magnetometers, goniometers, and force sensors, enable gait assessment under more natural and field-based conditions (12).

Inertial measurement units (IMUs) address several limitations of conventional gait evaluation systems. These electronic devices integrate accelerometers, gyroscopes, and magnetometers to measure acceleration, angular velocity, and orientation, thereby providing real-time information about the dynamic state of the body (13). IMUs have demonstrated excellent agreement with conventional motion-analysis systems, such as force platforms and pressure walkways (14, 15), while offering the advantage of field portability. A notable advantage of IMU-based analysis is its ability to identify compensatory lameness adaptations that extend beyond the primarily affected limb. For example, IMUs can detect head movement asymmetry, typically associated with forelimb lameness but also seen with hindlimb problems, and pelvic motion asymmetry, which is most strongly linked to hindlimb lameness (16, 17). This overlap means that head movement asymmetry may not specifically localize the affected limb, which can contribute to low interobserver agreement and misidentification, especially among inexperienced evaluators (18, 19). Recent studies in elephants have employed IMUs to investigate gait transition and spatiotemporal parameters (20–24). However, the limited availability of species-specific gait data from clinically sound elephants has constrained the interpretation of abnormal gait patterns. Establishing preliminary observed values for kinematic parameters therefore provides a foundation for the future clinical application of IMU-based gait analysis. This study aimed to report preliminary observed values for IMU-derived kinematic parameters in clinically sound Asian elephants and to evaluate their application for describing gait patterns in individual elephants with clinically observed abnormalities.

## 2 Materials and methods

### 2.1 Animals

This study was approved by the Institutional Animal Care and Use Committee, Faculty of Veterinary Medicine, Chiang Mai University, Chiang Mai, Thailand (license number S18/2567). Permission to use the elephants in the study was obtained from the owners.

Twelve clinically sound Asian elephants (*Elephas maximus*) were enrolled from the National Elephant Institute, Forest Industry Organization, and Taweechai Elephant Camp, Thailand. Each elephant received a physical examination performed by an experienced veterinarian to confirm the absence of musculoskeletal or neurological abnormalities. Visual gait assessments were performed independently by three veterinarians with more than 5 years of clinical experience in elephant healthcare. Assessments were based on consensus among all three assessors. The Gait Score system ranged from 0 to 2 (25): 0 indicated no visible lameness or limb abnormality, 1 indicated mild lameness or subtle gait alteration, and 2 indicated severe lameness characterized by evident gait abnormalities or limb deformities, including an unwillingness to flex, extend, or bear weight on the affected limb, additional descriptive clinical labels were recorded for abnormal cases. Elephants were excluded from final analysis if IMU signal quality was inadequate for reliable stance–swing phase detection or if the IMU dataset was incomplete for the predefined trial selection.

In addition, four Asian elephants with clinically identified abnormal gait patterns and a gait score of 2/2 based on consensus among all three assessors were independently enrolled from elephant camps in Thailand for descriptive case comparison. These elephants were not included in the clinically sound dataset. Instead, each case was evaluated individually and compared descriptively with the observed values from the clinically sound group to illustrate how IMU-derived metrics varied in clinically observed abnormal gait presentations.

### 2.2 Data collection

**Figure 1:**
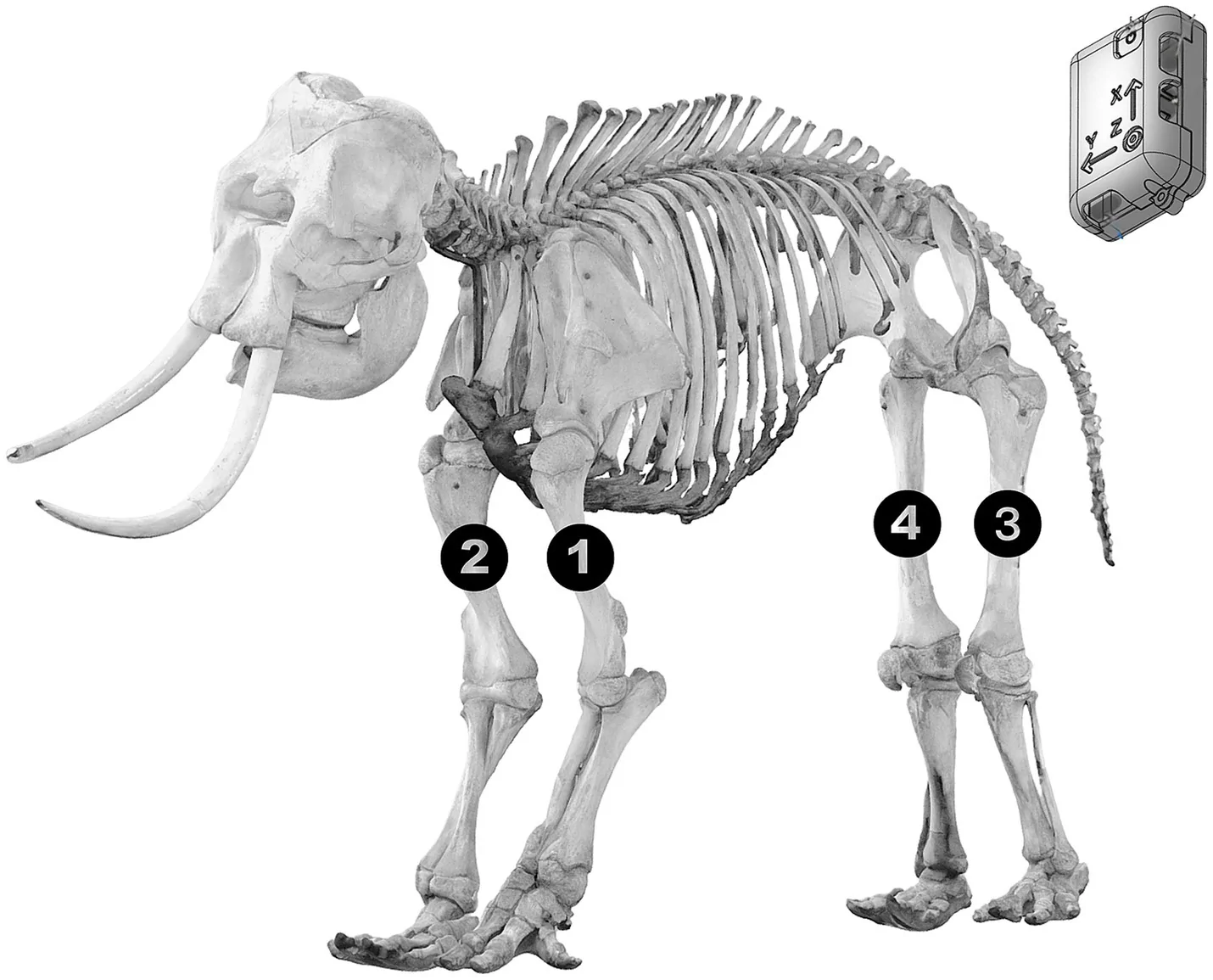
Placement of the four inertial measurement unit (IMU) sensors on the elephant skeleton. Sensors were positioned over the midshaft of the humerus and femur bilaterally to capture temporospatial and vertical displacement data. Each sensor recorded triaxial acceleration and angular velocity at 50 Hz.

Temporospatial parameters and vertical displacement of gait were measured with four inertial measurement unit (IMU) sensors (STT Ingeniera Y Sistemas, San Sebastián, Spain), each comprising an accelerometer, gyroscope, and magnetometer, sampling at 50 Hz. The sensors were securely attached to the skin overlying the midshaft of the humerus and femur bilaterally using hypoallergenic adhesive tape to minimize movement artifacts. Sensor alignment was checked visually relative to anatomical landmarks and adjusted as necessary. Figure 1 illustrates sensor placement.

The IMU system recorded triaxial acceleration and angular velocity (X, Y, and Z axes), providing six degrees of freedom data for gait analysis. Sensor signals were transmitted wirelessly via Wi-Fi to a software interface, and acceleration and deceleration effects were minimized through the standardized walkway design described below. Up to four consecutive trials were attempted per elephant, and data from up to three trials with the most consistent walking patterns were selected for analysis. Consistency was assessed based on continuous straight-line walking without stopping and maintenance of a steady pace throughout each trial. Among the 10 elephants included in the final analysis, nine completed three valid trials, and one completed two valid trials.

**Figure 2:**
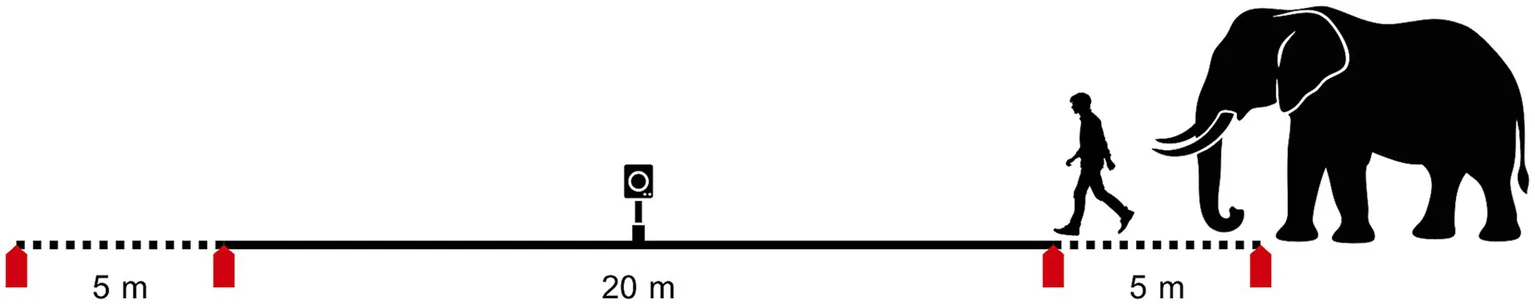
Schematic of the data collection setup. Each trial consisted of a 30-m walkway, including a 5-m acceleration zone, a 20-m data collection zone, and a 5-m deceleration zone. A GoPro Hero 10 camera (1440p, 240 fps) was placed at the center of the walkway to record high-speed video for gait-phase verification. Only data from the central 20 m were used for analysis to minimize the effects of acceleration and deceleration.

Prior to data collection, each elephant was familiarized with the testing environment and guided by its mahout using positive reinforcement techniques (food rewards and verbal cues) to maintain a steady walking pace. To minimize the confounding effect of walking speed on temporospatial timing parameters, elephants performed trials within a predefined speed range of 1.00 ± 0.20 m/s, selected based on preliminary testing as representative of a comfortable walking pace. A standardized walkway protocol was used to achieve stable, steady-state walking. To minimize the effects of acceleration and deceleration, each trial incorporated a 5-m approach zone before entering the measurement area and a 5-m exit zone after leaving it. Only trials with a mean walking speed within 0.80–1.20 m/s and without stopping or deviation from a straight path were retained for analysis. Walking speed was calculated from IMU-derived trial duration as the 30-m walkway distance divided by the time elapsed from the start to the end of each trial. Although speed screening was based on the full 30-m trial, gait-cycle extraction and subsequent analyses were restricted to the central 20 m to reduce acceleration and deceleration effects. Each elephant walked a total of 30 m per trial, with gait data collected only within the central 20-m section, where velocity was most stable (Figure 2). A GoPro Hero 10 camera (1440p, 240 fps) was positioned at the midpoint of the walkway to obtain high-speed video recordings synchronized with IMU data. These recordings were used to visually verify stance and swing phase identification for each limb during signal processing. All trials were performed on level hard ground (concrete or asphalt) to ensure consistent surface conditions.

### 2.3 Signal processing and data analysis

IMU data were imported into custom MATLAB scripts developed for vertical movement analysis. Vertical acceleration signals were filtered using a 4th-order Butterworth band-pass filter with cutoff frequencies of 0.3 and 6.0 Hz (26, 27). This cutoff was selected to remove integration drift from accelerometer bias while preserving gait-cycle frequency components. Filtered acceleration signals were then double integrated to obtain displacement over time, and displacement was expressed in millimeters.

Five consecutive gait cycles were selected from the middle portion of each trial, ensuring data stability and excluding acceleration or deceleration phases. Temporospatial parameters were quantified using two parallel analytical approaches. Absolute timing was reported in seconds, whereas normalized timing was expressed as a percentage of the gait cycle to facilitate speed-independent comparisons. Reporting both formats preserves natural temporal features of locomotion while enabling speed-independent assessment of gait phase proportions.

Temporal parameters were extracted from the non-normalized displacement signals for all four limbs, including the left forelimb, right forelimb, left hindlimb, and right hindlimb. The outcomes included stride time, stance time, swing time, and the corresponding stance and swing phase percentages, and values were averaged across five consecutive gait cycles. Stance and swing were defined as the fractions of each stride spent in ground contact and non-contact, respectively. The beginning of each gait cycle was referenced to the minimum displacement point, which corresponded to stance onset. Concurrent high-speed video recordings were reviewed frame-by-frame to confirm gait-event detection and to verify stance and swing classification. The duty factor (DF) was calculated as the ratio of stance time to total gait cycle duration.

To minimize the influence of velocity differences among trials, gait cycles were time-normalized to a 0–1 interval before further processing. Cross-correlation analysis was performed following a previously validated IMU-based protocol (24) to evaluate bilateral vertical movement similarity between the left and right limbs. Cross-correlation coefficients (COF) were calculated from the time-normalized direct vertical displacement waveforms (mm) by computing the normalized cross-correlation between the averaged left and right displacement curves over a range of time lags and extracting the peak correlation value. COF therefore reflects similarity between left and right limb vertical displacement waveforms, with values closer to +1 indicating greater similarity in movement patterns between sides. All analyses were performed using custom MATLAB scripts developed in the pilot study to identify and compare bilateral vertical movement similarity for each left–right limb pair.

### 2.4 Statistical analysis

Normality was evaluated using the Shapiro–Wilk test. Because the sample size was small (*n* = 10) and at least one outcome showed clear deviation from normality, paired left–right comparisons of temporospatial parameters were performed using the Wilcoxon signed-rank test (two-tailed) to maintain a consistent analytical approach. For each elephant, outcome values were averaged across all valid trials (two trials for one elephant and three trials for nine elephants) prior to left–right comparison. Statistical significance was set at *p* < 0.05. These comparisons were considered exploratory, and no adjustment for multiple testing was applied.

## 3 Results

### 3.1 Gait characteristics in clinically sound elephants

All twelve elephants met the inclusion criteria for sound locomotion based on agreement among all three assessors. The primary challenge during data collection was ensuring that elephants walked in a straight line at a steady pace without interruptions. Two elephants were excluded from analysis due to unusable or incomplete IMU datasets, resulting in a final analyzed cohort of ten elephants (*n* = 10). All retained trials (*n* = 29 trials from 10 elephants) met the predefined walking-speed criterion (0.80–1.20 m/s), with a mean velocity per elephant of 0.93 ± 0.08 m/s (range 0.82–1.06 m/s across individuals). Gait-cycle extraction was restricted to the central 20 m of the walkway to minimize acceleration and deceleration effects.

Morphometric measurements for the ten elephants included in the final analysis showed shoulder heights ranging from 220 to 276 cm (mean 242.40 ± 16.83 cm). The proximal forelimb segment measured 62–90 cm (mean 78.30 ± 10.48 cm), while the distal forelimb segment measured 91–123 cm (mean 101.40 ± 9.88 cm). The proximal hindlimb segment ranged from 74 to 100 cm (mean 85.30 ± 7.99 cm), and the distal hindlimb segment ranged from 67 to 92 cm (mean 78.30 ± 7.41 cm).

Exploratory Wilcoxon signed-rank tests, using per-elephant means from two to three valid trials, were used to evaluate left–right limb differences across gait parameters (Table 1). For each trial, temporospatial parameters were derived from five consecutive gait cycles selected from the middle portion of the trial to ensure data stability. In the forelimbs, significant side differences were detected for stance time (*Z* = −1.992, *p* = 0.046), stance phase percentage (*Z* = −2.090, *p* = 0.037), and swing phase percentage (*Z* = −2.090, *p* = 0.037), with greater right-side stance allocation and reduced right swing phase. No significant differences were observed for forelimb stride time (*Z* = −0.106, *p* = 0.915), and forelimb swing time showed a borderline difference (*Z* = −1.939, *p* = 0.052).

**Table 1:** Comparisons of left and right temporospatial gait parameters (*n* = 10 elephants).

Parameter	Mean left ± SD	Median left (IQR)	Mean right ± SD	Median right (IQR)	*Z*	*p*-value*
Forelimb						
Stride Time (s)	2.07 ± 0.23	2.18 (0.40)	2.08 ± 0.22	2.16 (0.38)	−0.106	0.92
Stance Time (s)	1.50 ± 0.24	1.42 (0.34)	1.56 ± 0.23	1.51 (0.17)	−1.992	<0.05*
Stance Phase (%)	72.30 ± 7.33	71.09 (10.28)	75.29 ± 7.20	74.38 (8.37)	−2.090	<0.05*
Swing Time (s)	0.57 ± 0.17	0.55 (0.25)	0.51 ± 0.17	0.56 (0.25)	−1.939	0.05†
Swing Phase (%)	27.70 ± 7.33	28.92 (10.28)	24.71 ± 7.20	25.63 (8.37)	−2.090	<0.05*
Hindlimb						
Stride time (s)	2.06 ± 0.21	2.10 (0.38)	2.07 ± 0.21	2.15 (0.38)	−0.680	0.50
Stance time (s)	1.42 ± 0.14	1.39 (0.21)	1.51 ± 0.10	1.51 (0.14)	−2.490	<0.05*
Stance phase (%)	69.16 ± 3.98	70.43 (5.80)	73.29 ± 4.71	71.61 (5.86)	−2.599	<0.05*
Swing time (s)	0.64 ± 0.13	0.66 (0.19)	0.56 ± 0.14	0.63 (0.19)	−2.705	<0.05*
Swing phase (%)	30.84 ± 3.98	29.58 (5.80)	26.71 ± 4.71	28.40 (5.86)	−2.599	<0.05*

*Wilcoxon signed-rank test, two-tailed. Bold *p*-values indicate statistical significance at *p* < 0.05. SD, standard deviation; IQR, interquartile range; L, left; R, right; *Z* = Wilcoxon signed-rank test statistic. ^†^Forelimb swing time: *p* = 0.052; not significant at *p* < 0.05.

In the hindlimbs, stance-related parameters showed significant side differences. Right hindlimb stance time was greater than the left (*Z* = −2.490, *p* = 0.013) and stance phase percentage was higher on the right side (*Z* = −2.599, *p* = 0.009). Correspondingly, right hindlimb swing time (*Z* = −2.705, *p* = 0.007) and swing phase percentage (*Z* = −2.599, *p* = 0.009) were lower than the left. No significant difference was observed for hindlimb stride time (*Z* = −0.680, *p* = 0.496).

Duty factor analysis showed slightly higher right-side values in both forelimb and hindlimb pairs, consistent with the stance-phase patterns described above (Table 2). Cross-correlation analysis showed high bilateral vertical movement similarity in both forelimb and hindlimb pairs in clinically sound elephants (Table 3).

**Table 2:** Duty factor (DF) for forelimb and hindlimb gait (*n* = 10 elephants).

Limb	DF_L_	DF_R_
Forelimb	0.72 ± 0.07	0.75 ± 0.07
Hindlimb	0.69 ± 0.04	0.73 ± 0.05

DF: proportion of the gait cycle spent in stance phase.

**Table 3:** Cross-correlation coefficient (COF) describing bilateral vertical movement similarity in forelimb and hindlimb pairs.

Limb pair	Minimum	Maximum	Mean ± SD
Forelimb	0.88	0.99	0.93 ± 0.04
Hindlimb	0.93	1.00	0.97 ± 0.02

### 3.2 Descriptive comparison with clinically abnormal gait cases

Because the abnormal cases represented heterogeneous clinical presentations, affected limbs, and clinically observed pain-related signs, they were not pooled for inferential comparison with the clinically sound elephant group. Gait data from four elephants with a clinical gait score of 2/2 are presented in Table 4. These cases were used as individual illustrative examples and were not intended to characterize diagnostic categories or underlying mechanisms.

**Table 4:** Descriptive comparison of gait parameters and bilateral vertical movement similarity between clinically sound elephants and four clinically abnormal gait cases.

Parameter	Clinically sound elephants		A1		A2		A3		A4	
	Left limb (Mean ± SD)	Right limb (Mean ± SD)	Left limb (Mean ± SD)	Right limb (Mean ± SD)	Left limb (Mean ± SD)	Right limb (Mean ± SD)	Left limb (Mean ± SD)	Right limb (Mean ± SD)	Left limb (Mean ± SD)	Right limb (Mean ± SD)
Forelimb										
Stance (%)	72.30 ± 7.33	75.29 ± 7.20	71.21 ± 3.88	81.70 ± 3.05	78.18 ± 2.39	70.75 ± 2.77	77.85 ± 4.68	76.88 ± 5.65	62.08 ± 1.35 ↓	60.95 ± 5.16 ↓
Swing (%)	27.70 ± 7.33	24.71 ± 7.20	28.79 ± 3.88	18.30 ± 3.05	21.82 ± 2.39	29.25 ± 2.77	22.15 ± 4.68	23.12 ± 5.65	37.92 ± 1.35 ↑	39.05 ± 5.16 ↑
DF	0.72 ± 0.07	0.75 ± 0.07	0.71 ± 0.04	0.82 ± 0.03	0.78 ± 0.02	0.71 ± 0.03	0.78 ± 0.05	0.77 ± 0.06	0.62 ± 0.01 ↓	0.61 ± 0.05 ↓
COF	0.93 ± 0.04		0.76 ± 0.03 ↓		0.97 ± 0.02		0.72 ± 0.04 ↓		0.98 ± 0.01	
Hindlimb										
Stance (%)	69.16 ± 3.98	73.29 ± 4.71	62.47 ± 1.65 ↓	60.89 ± 2.82 ↓	59.39 ± 4.03 ↓	57.62 ± 2.63 ↓	64.03 ± 4.30 ↓	64.36 ± 7.28 ↓	76.16 ± 1.24 ↑	61.78 ± 0.28 ↓
Swing (%)	30.84 ± 3.98	26.71 ± 4.71	37.53 ± 1.65 ↑	39.11 ± 2.82 ↑	40.61 ± 4.03 ↑	42.38 ± 2.63 ↑	35.97 ± 4.30 ↑	35.64 ± 7.28 ↑	23.84 ± 1.24 ↓	38.22 ± 0.28 ↑
DF	0.69 ± 0.04	0.73 ± 0.05	0.63 ± 0.02 ↓	0.61 ± 0.03 ↓	0.59 ± 0.04 ↓	0.58 ± 0.03 ↓	0.64 ± 0.04 ↓	0.64 ± 0.07 ↓	0.76 ± 0.01 ↑	0.62 ± 0.00 ↓
COF	0.97 ± 0.02		0.85 ± 0.03 ↓		0.98 ± 0.01		0.83 ± 0.06 ↓		0.88 ± 0.05 ↓	

A1 = clinically observed pain-associated left forelimb gait abnormality with circumduction; A2 = chronic right forelimb structural deformity without clinically observable pain-related signs; A3 = clinically observed pain-associated left hindlimb gait abnormality with circumduction; A4 = chronic left hindlimb structural deformity without clinically observable pain-related signs. Values are mean ± SD. ↑ and ↓ indicate values above or below the clinically sound mean ± SD interval, respectively; for COF, only ↓ is shown to indicate reduced bilateral vertical movement similarity. Symbols are descriptive and do not represent diagnostic thresholds or clinically relevant cut-off values.

A1 presented with a clinically observed pain-associated left forelimb gait abnormality with circumduction. Compared with the observed values in clinically sound elephants, A1 showed a higher right forelimb DF and lower forelimb and hindlimb COF values.

A2 presented with chronic right forelimb structural deformity without clinically observable pain-related signs during clinical assessment. Compared with the observed values in clinically sound elephants, A2 showed forelimb and hindlimb COF values within the clinically sound mean ± SD interval. Hindlimb DF values were lower bilaterally compared with the clinically sound group means.

A3 presented with a clinically observed pain-associated left hindlimb gait abnormality with circumduction. Compared with the observed values in clinically sound elephants, A3 showed lower forelimb and hindlimb COF values and reduced hindlimb DF values bilaterally.

A4 presented with chronic left hindlimb structural deformity without clinically observable pain-related signs during clinical assessment. Compared with the observed values in clinically sound elephants, A4 showed marked hindlimb DF asymmetry, with a higher DF on the left hindlimb than the right hindlimb. Forelimb COF was not below the clinically sound mean ± SD interval, whereas hindlimb COF was below this interval.

**Figure 3:**
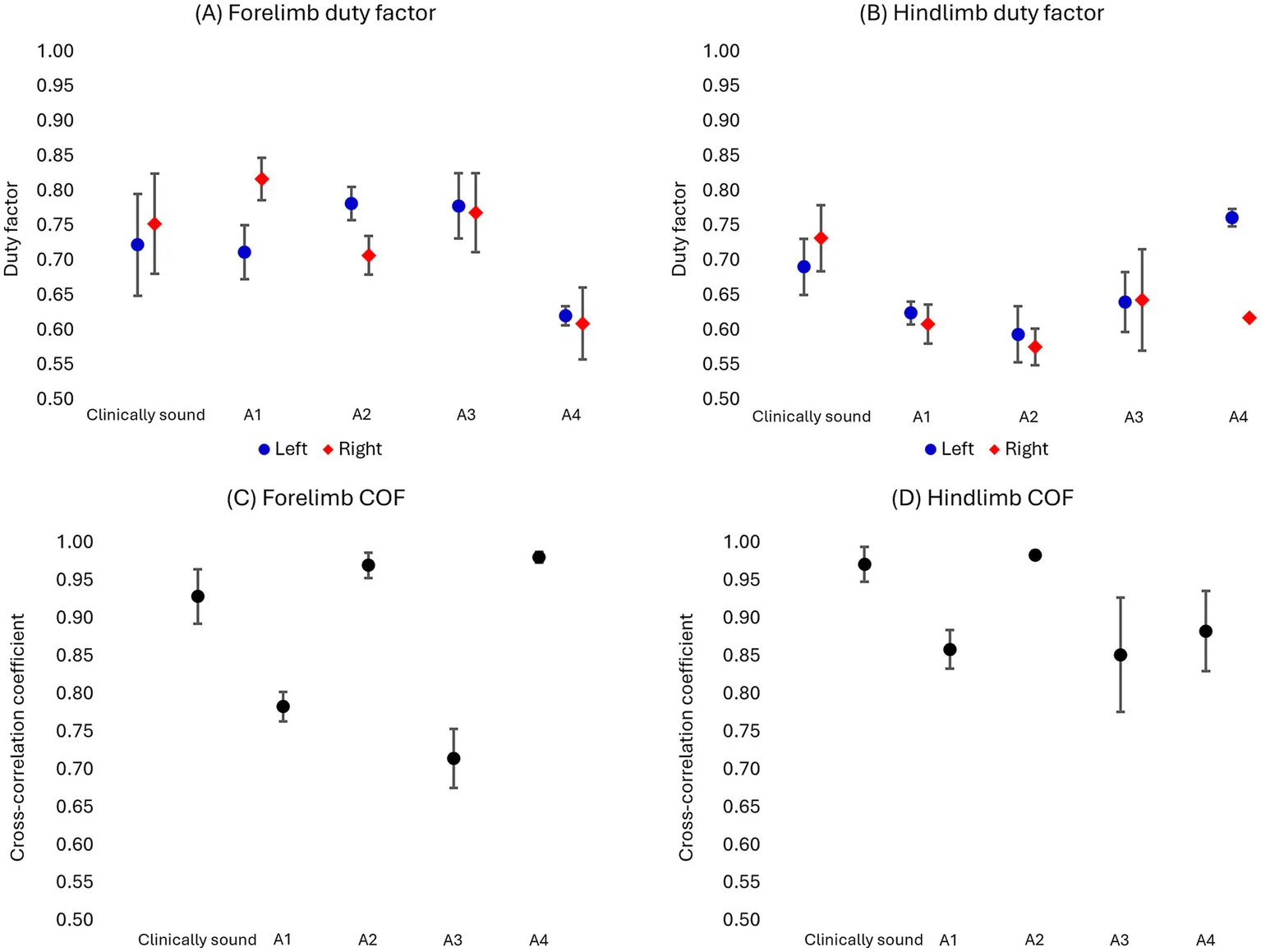
Graphical comparison of duty factor (DF) and cross-correlation coefficient (COF) values between clinically sound elephants and four clinically abnormal gait cases. DF values are shown for the left and right forelimbs **(A)** and hindlimbs **(B)**, and COF values for forelimb **(C)** and hindlimb **(D)** left–right limb pairs. Clinically sound values are presented as mean ± SD from 10 elephants, whereas abnormal cases are presented descriptively as mean ± SD from repeated trials. A1 = clinically observed pain-associated left forelimb gait abnormality with circumduction; A2 = chronic right forelimb structural deformity without clinically observable pain-related signs; A3 = clinically observed pain-associated left hindlimb gait abnormality with circumduction; A4 = chronic left hindlimb structural deformity without clinically observable pain-related signs.

Overall, the four abnormal cases showed individual combinations of altered DF and COF values. These were individual descriptive observations, were not subjected to inferential statistical comparisons, and should not be interpreted as evidence of group or mechanistic differences. Figure 3 presents descriptive graphical comparisons of DF and COF values between clinically sound elephants and the individual abnormal gait cases.

## 4 Discussion

The present study provides new quantitative insight into gait mechanics in Asian elephants by documenting preliminary observed values for gait parameters and demonstrating how these metrics may help contextualize clinically identified abnormal gait patterns relative to observed values in clinically sound elephants. Patterns in the temporospatial parameters and IMU-derived vertical displacement illustrate how elephants modify bilateral movement patterns and stance allocation during altered locomotion. Integrating IMU-derived indicators with clinical assessment may help identify deviations that are difficult to detect visually and support the future development of species-specific tools for gait evaluation in elephant veterinary medicine. Although IMUs have been applied to capture objective gait data in elephants (20–22, 28), interpretation has been constrained by limited species-specific data from clinically sound elephants. In the present study, temporospatial parameters and symmetry-related metrics were quantified in clinically sound elephants to provide preliminary observed values, and these values were used descriptively to contextualize four abnormal gait cases involving both forelimb and hindlimb presentations.

Temporospatial parameters in clinically sound elephants demonstrated consistent stride timing across limbs, with small left–right differences in stance and swing phases. Notably, stance allocation showed a small but consistent right-side bias in this clinically sound group, with greater right stance time and stance phase and correspondingly reduced right swing phase, while stride time remained similar between sides. This pattern should be considered when interpreting left–right differences in abnormal gait cases.

Several factors may contribute to this side-related pattern. Prior biomechanical studies indicate that elephants may exhibit mild inherent asymmetry related to lateralized limb function and stable lateral-sequence gait patterns (29). Symmetry indices, including COF measures, were consistent with high bilateral vertical movement similarity and minor inherent asymmetries, which may reflect natural limb dominance or baseline laterality rather than pathological deviation, comparable to patterns described in horses (30, 31).

In addition, all clinically sound elephants in the present study were captive elephants in Thailand and were managed under the routine Thai mahout-guided handling system, in which elephants are habitually controlled from the elephant’s right side, corresponding to the mahout’s left side. Because this handling pattern was uniform across all elephants in the present study, it could not be formally tested as an explanatory variable within the current dataset. Therefore, handling laterality should be interpreted as a potential population-specific factor rather than a confirmed cause of the observed asymmetry.

The direction of this routine handling practice is consistent with the left–right differences observed in the present dataset, although a causal relationship cannot be confirmed. Repeated performance of side-specific commands may contribute to habitual limb-use preferences, similar to training-associated laterality documented in domestic dogs and horses (32, 33). Elephants typically support a greater proportion of body weight on the forelimbs, approximately 55–60%, which may contribute to small temporal asymmetries during normal locomotion (34). Overall, these findings emphasize the need to account for normal side-related variation when interpreting IMU-derived gait parameters in elephants with abnormal gait patterns.

Previous studies in elephant biomechanics provide important context for the present findings. Earlier work by Hutchinson et al. and Ren et al. established foundational kinematic and locomotor biomechanics data for elephants using video-based motion analysis and force-platform approaches (23, 29). These studies provided important information on elephant locomotion, speed-related gait characteristics, and whole-body mechanics. However, these methods differ from the field-deployable IMU approach used in the present study and were not designed to provide side-specific IMU-derived comparisons of stance allocation and bilateral vertical movement similarity within forelimb and hindlimb pairs.

Our previous IMU-based elephant gait studies demonstrated the feasibility of wearable sensor-based gait assessment, described gait variability in lame elephants, and applied cross-correlation analysis to evaluate vertical movement symmetry in a small pilot sample (20, 22, 24). The present study extends this earlier work by applying a standardized field-based walking protocol to clinically sound elephants and by reporting preliminary observed values across temporospatial parameters, DF, and COF. In addition, the present study provides side-specific assessment of DF for individual limbs and limb-pair assessment of bilateral vertical movement similarity using COF, allowing descriptive comparison between clinically sound elephants and multiple abnormal gait cases. This approach provides a structured framework for interpreting stance allocation and bilateral movement-pattern adaptations beyond single-case description or feasibility-focused IMU assessment.

Compensatory gait patterns may be interpreted using principles comparable to the “law of sides” described in equine lameness studies (35). In horses, a primary lame limb is offloaded, the ipsilateral limb may show reduced loading, and the contralateral limb often assumes increased loading as part of a coordinated redistribution strategy (36, 37), with comparable patterns documented particularly during trot (38–40). However, elephants are evaluated during walking with variable two- and three-limb support phases, so load redistribution may involve whichever limbs are concurrently loaded rather than a single consistent diagonal strategy. Elephant locomotion is further constrained by substantial body mass and high stability requirements and is characterized by low-amplitude center-of-mass vertical motion and synchronized limb dynamics (23, 41). Accordingly, compensatory mechanisms may prioritize overall balance through temporal reallocation and altered bilateral vertical movement patterns rather than large unilateral load transfer.

The four abnormal gait cases illustrated how COF varied among individual clinical presentations when interpreted descriptively against the values observed in clinically sound elephants. A1 and A3 each showed lower COF values in both forelimb and hindlimb pairs despite their different clinical presentations. A2 showed COF values within the clinically sound mean ± SD interval in both limb pairs. A4 showed a different combination, with forelimb COF not below this interval but lower hindlimb COF. These observations describe four clinically heterogeneous cases and should not be interpreted as differences between pain-associated gait abnormalities and structural deformities or as evidence of distinct underlying mechanisms.

DF patterns varied according to the affected limb and case presentation. A1 showed the most evident right forelimb DF increase, whereas A4 showed the most pronounced hindlimb DF asymmetry. A3 showed reduced hindlimb DF bilaterally rather than a strong unilateral difference. These patterns indicate that DF may help describe stance allocation changes, but apparent left–right similarity does not necessarily indicate normal gait if both limbs within a pair shift in the same direction.

Taken together, these findings support the descriptive use of DF and COF as complementary IMU-derived metrics. However, diagnoses were not confirmed by diagnostic imaging, and the abnormal cases were few and heterogeneous. Therefore, these observations should be interpreted as exploratory descriptions of individual cases rather than evidence of group differences, confirmed mechanisms, or clinically relevant thresholds. Future studies with larger sample sizes, confirmed diagnoses, controlled speed conditions, repeated testing sessions, and standardized test–retest designs are needed to determine the repeatability of IMU-derived parameters and to evaluate whether these metrics can support clinical gait assessment in elephants.

Several limitations should be considered when interpreting the findings. The clinically sound group consisted of only ten elephants, and the abnormal case series included only four clinically heterogeneous individuals. Two cases had clinically observed pain-associated gait abnormalities, and two had structural deformities without clinically observable pain-related signs. However, these descriptions were based on clinical observation and physical examination and were not treated as objectively confirmed diagnostic categories. The cases involved both forelimb and hindlimb regions, limiting the generalizability of the findings and precluding interpretation of differences between the two clinical presentations. The specific pathologies underlying the gait abnormalities were not confirmed through diagnostic imaging, such as radiography or ultrasonography. Factors such as injury type, pain severity, and chronicity therefore could not be evaluated. In addition, although walking speed was controlled within a defined range, residual variation in speed and trial selection may still have influenced temporospatial metrics.

Technical limitations were inherent to the use of IMUs. In the present study, sensors were attached only over the humerus and femur regions; therefore, the analysis focused on forelimb and hindlimb vertical displacement derived from these sensor locations. Distal limb movement was not evaluated, and future studies incorporating additional IMUs over distal limb segments may provide further insight into limb-segment coordination, joint-level compensation, and localized movement adaptations associated with different types of gait abnormality. The calculation of vertical displacement required double integration of acceleration data, which can be susceptible to drift. In addition, the synchronization between video recordings and sensor signals was not fully aligned, and a time lag remained during gait phase identification in each trial. DF and COF showed potential for characterizing gait asymmetry and bilateral vertical movement similarity, but these metrics require further validation before diagnostic thresholds can be established. Threshold values for clinically relevant deviations have not yet been confirmed in larger samples, highlighting the need for broader clinical testing across a range of lameness severities and types.

In conclusion, this study provided preliminary observed values for IMU-derived gait parameters in clinically sound Asian elephants and demonstrated how DF and COF can be used to describe individual abnormal gait presentations. Across four clinically heterogeneous cases, these metrics identified case-specific combinations of altered stance allocation and bilateral vertical movement similarity, supporting their complementary use in objective gait characterization. These individual observations should not be interpreted as evidence of differences between pain-associated gait abnormalities and structural deformities or as indicators of distinct underlying mechanisms. Despite these limitations, the findings demonstrate the potential value of IMU-based gait analysis for providing quantitative information that complements clinical assessment in elephants. Larger clinically characterized datasets with objective diagnostic confirmation are needed to define clinically relevant thresholds and evaluate clinical applicability.

## Data Availability

The raw data supporting the conclusions of this article will be made available by the authors, without undue reservation.
